# Effects of different hinge positions on tibial rotation in uniplanar medial opening wedge high tibial osteotomy with three-dimensional tibial models

**DOI:** 10.3389/fsurg.2024.1441777

**Published:** 2024-10-30

**Authors:** Lizhong Jing, Yulian Ren, Shaoshan Wang, Jiushan Yang, Jian Wang

**Affiliations:** ^1^ Department of Orthopaedic Surgery, Shandong Provincial Hospital, Shandong University, Jinan, Shandong Province, China; ^2^ Department of Orthopedics, The Affiliated Hospital of Shandong University of Traditional Chinese Medicine, Jinan, Shandong Province, China; ^3^ Department of Infection Management, The Affiliated Hospital of Shandong University of Traditional Chinese Medicine, Jinan, Shandong Province, China

**Keywords:** high tibial osteotomy, varus deformity, hinge axis, 3D model, distal tibial rotation

## Abstract

**Background:**

To investigate the effects of different hinge positions in the sagittal and axial planes on distal tibial rotation (DTR) during medial opening wedge high tibial osteotomy (MOWHTO) with three-dimensional tibial models.

**Methods:**

Preoperative CT data from 30 knee joints in 30 patients who underwent surgery for varus malalignment of knee were included. 1 standard hinge position (0°), 6 axial planes (±5°, ±10°, ±15°), and 6 sagittal planes (±5°, ±10°, ±15°) hinge positions were defined and virtual uniplanar osteotomy was performed. The correction angle of each model was generated using Fujisawa's point. Participants’ baseline characteristics, radiologic parameters and DTR were measured. One-Way Repeated Measures ANOVA and single factor linear regression analysis were used to analyze the association between tibial rotation and hinge position in the sagittal and axial planes.

**Results:**

We found a clear linear correlation between changes in hinge position in the sagittal plane and DTR. The changes in DTR were the smallest when the hinge position was at 5°, where internal or external rotation of the DTR may occur. When the front aspect of hinge axis rotated distally, DTR tended towards internal. Meanwhile, when the front aspect of hinge axis rotated proximally, DTR tended towards external. There were no correlations with every hinge axis position in the axial plane.

**Conclusions:**

It is sagittal but not axial hinge axis affects DTR in uniplanar MOWHTO with three-dimensional tibial models. In the sagittal plane, every change in hinge position was significantly linearly correlated with DTR. However, no linear correlations were observed between every hinge position change in the axial plane.

## 1 Introduction

Medial opening wedge high tibial osteotomy (MOWHTO) is the primary surgical option for active young patients with varus malalignment of the knee. By correcting the varus malalignment, the pressure and pain of the medial compartment of the knee joint can be reduced (1).

In addition to correcting coronal alignment, MOWHTO can unintentionally influence the Posterior Tibial Slope (PTS) in the sagittal plane and distal tibial rotation (DTR) relative to the proximal tibia in the axial plane due to the triangular shape of the proximal tibia (2–4). To date, relevant studies have mostly focused on changes in the PTS. There is a lack of relevant studies investigating DTR during MOWHTO, and the conclusions drawn from existing studies are controversial.

One widely discussed factor responsible for the PTS change is hinge axis position, and the general belief is that an anterolateral axial hinge axis could reduce PTS while a posterolateral position could increase PTS (2, 5–7). But how DTR is influeced by hinge positions was still not fully understood. In our previous clinical research including 106 knees, we found there was different effects of hinge positions in the sagittal and axial planes on DTR during biplane MOWHTO (8). Obviously, it was just a qualitative research because of the existence of soft tissue around the knee. Considering adverse influences of tibial rotation on the contact pressures and kinematics of the knee and ankle (9, 10), further research is necessary.

The use of software to perform virtual osteotomy on a 3D model can eliminate the influence of complex factors such as soft tissue and allow for a more focused investigation of the bone structure, which is a reliable research method to assess the quantitative relationship between hinge positions and DTR change. Only two studies were found to have explored the impact of hinge axis angle on PTS during uniplanar MOWHTO using 3D surgical simulation studies (2, 5). The important finding was that the axial hinge position change significantly influences PTS, but the sagittal hinge position change has no effect. Notably, no study has explored the effect of different hinge positions on DTR by this method.

As such, the aim of the present study was to evaluate the effects of hinge positions in the sagittal and axial planes on DTR during MOWHTO, as well as to quantify the impact of hinge positions on DTR. A hypothesis was formulated that both sagittal and axial hinge positions would influence DTR during uniplanar MOWHTO, as demonstrated by 3D tibial models.

## 2 Materials and methods

### 2.1 Patients

From May 2020 to May 2021, 30 knee joints in 30 patients who underwent surgery for varus malalignment of the knee in our hospital were enrolled. Each patient underwent preoperative full-length lower-extremity CT and standing hip-to-ankle anteroposterior radiography. Every patient met the following inclusion criteria: symptomatic medial compartment of knee joint; varus malalignment over 4° between the tibial and femoral mechanical axis and medial proximal tibial angle (MPTA) less than 84° measured on a hip-to-ankle standing AP radiograph; osteoarthritis grades 0–2 of the medial compartment according to Kellgren and Lawrence. Exclusion criteria: a mechanical lateral distal femoral angle (mLDFA) >91° in the deformity analysis; severe varus malalignment considering the double level osteotomy (open wedge HTO and closed wedge distal femoral osteotomy); and severe medial compartment osteoarthritis (grades 3–4). Additionally, the Ethics Committee of our hospital gave approval to the present study, and every patient gave informed consent.

### 2.2 CT data and 3D bony geometry acquisition

All CT data were obtained by means of the same CT scanner (Revolution 256 CT; GE Healthcare, Milwaukee WI, USA). The slice thickness was 0.625 mm, and the acquisition matrix was 512 × 512 pixels. The DICOM (Digital Imaging and Communications in Medicine) data were downloaded using INFINITT PACS (INFINITT Healthcare, Seoul, Korea) and imported into Mimics (Materialise, Leuven, Belgium). The 3D models of the tibia and fibula were subsequently created. After the surface smoothness of the 3D models was processed using Geomagic Wrap (3D Systems Co. Ltd. USA) software, the bony geometry was imported into Geomagic Design X (Geomagic Inc., Morrisville, NC, USA) for the establishment of Coordinate System and virtual osteotomy, similar to the method described by Teng et al. (5).

### 2.3 The establishment of the coordinate system

**Figure 1:**
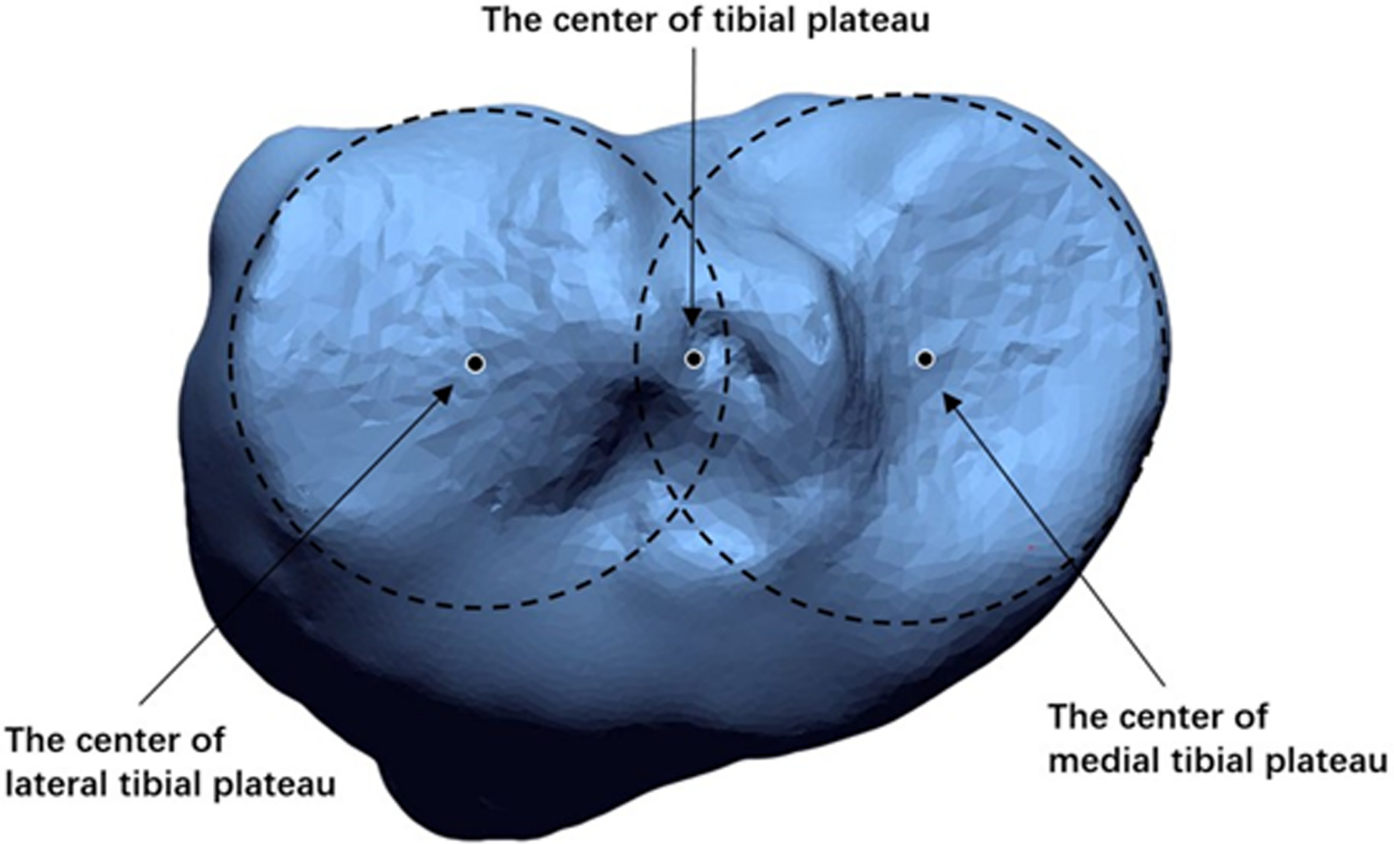
The center of the tibial plateau was determined as the midpoint of the centers of the lateral and medial plateaus of the tibia, which were manually determined using the method of the best-fit circle that covered both plateaus**.**

**Figure 2:**
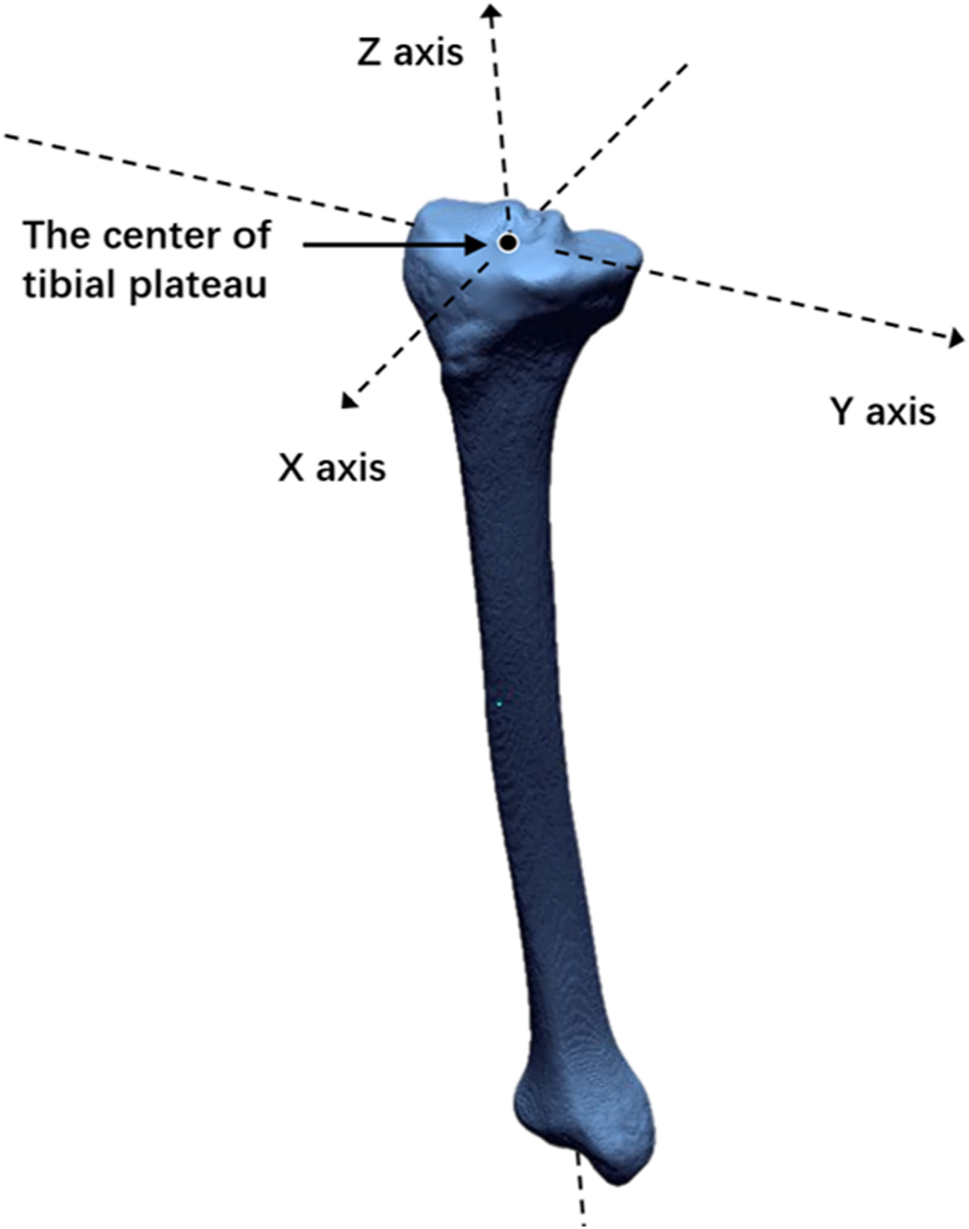
*Z*-axis is defined as the axis passing through the center of both the tibial plateau and ankle. *X*-axis is defined as the axis intersecting at right angles with the Z-axis and passing through the center of the tibial plateau, parallel to the Akagi line. *Y*-axis is defined as the axis intersecting at right angles with the *X*-axis and Z-axis through the center of the tibial plateau.

First, the *Z*-axis, also known as the tibial axis, was defined as the axis passing through the center of both the tibial plateau and ankle. The center of the tibial plateau was determined as the midpoint of the centers of the lateral and medial plateaus of the tibia, which were manually determined using the method of the best-fit circle that covered both plateaus (Figure 1). The center of the ankle was defined as the midpoint of the articular surface of the talar dome. Second, the *X*-axis was defined as the axis intersecting at right angles with the Z-axis and passing through the center of the tibial plateau, parallel to the Akagi line, which connects the middle of the posterior cruciate ligament (PCL) and the medial edge of the patellar tendon attachment (11). Third, the *Y*-axis was defined as the axis intersecting at right angles with the *X*-axis and Z-axis through the center of the tibial plateau. Finally, the XY plane, XZ plane, and YZ plane were defined, respectively (Figure 2).

### 2.4 Virtual osteotomy and hinge axis

**Figure 3:**
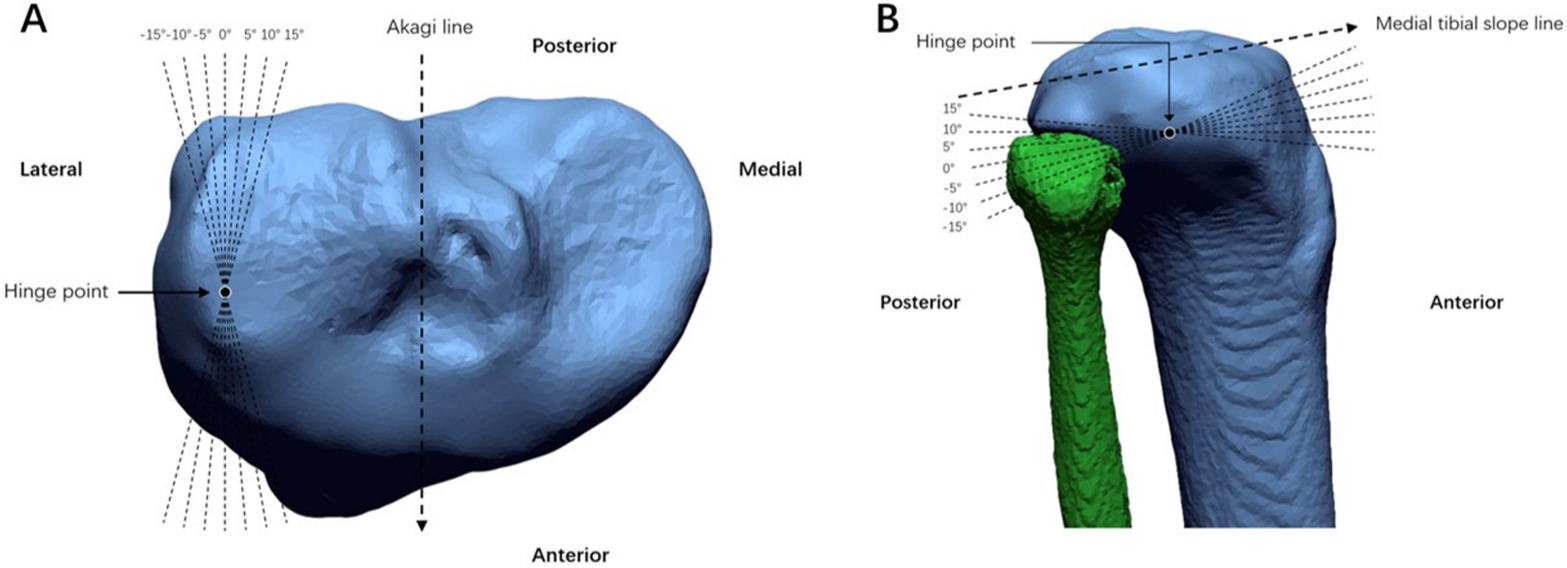
The standard hinge position (0°) was defined as hinge axis through the hinge point and parallel to the medial tibial slope line in the sagittal plane and akagi line in the axial plane. **(A)** 6 axial planes (±5°, ±10°, ±15°) hinge positions were defined as the hinge axis rotated internally and externally around the midpoint of the standard hinge axis in axial plane by different angle. **(B)** 6 sagittal planes (±5°, ±10°, ±15°) hinge positions were defined as hinge axis rotated proximally and distally in sagittal plane by different angle.

**Figure 4:**
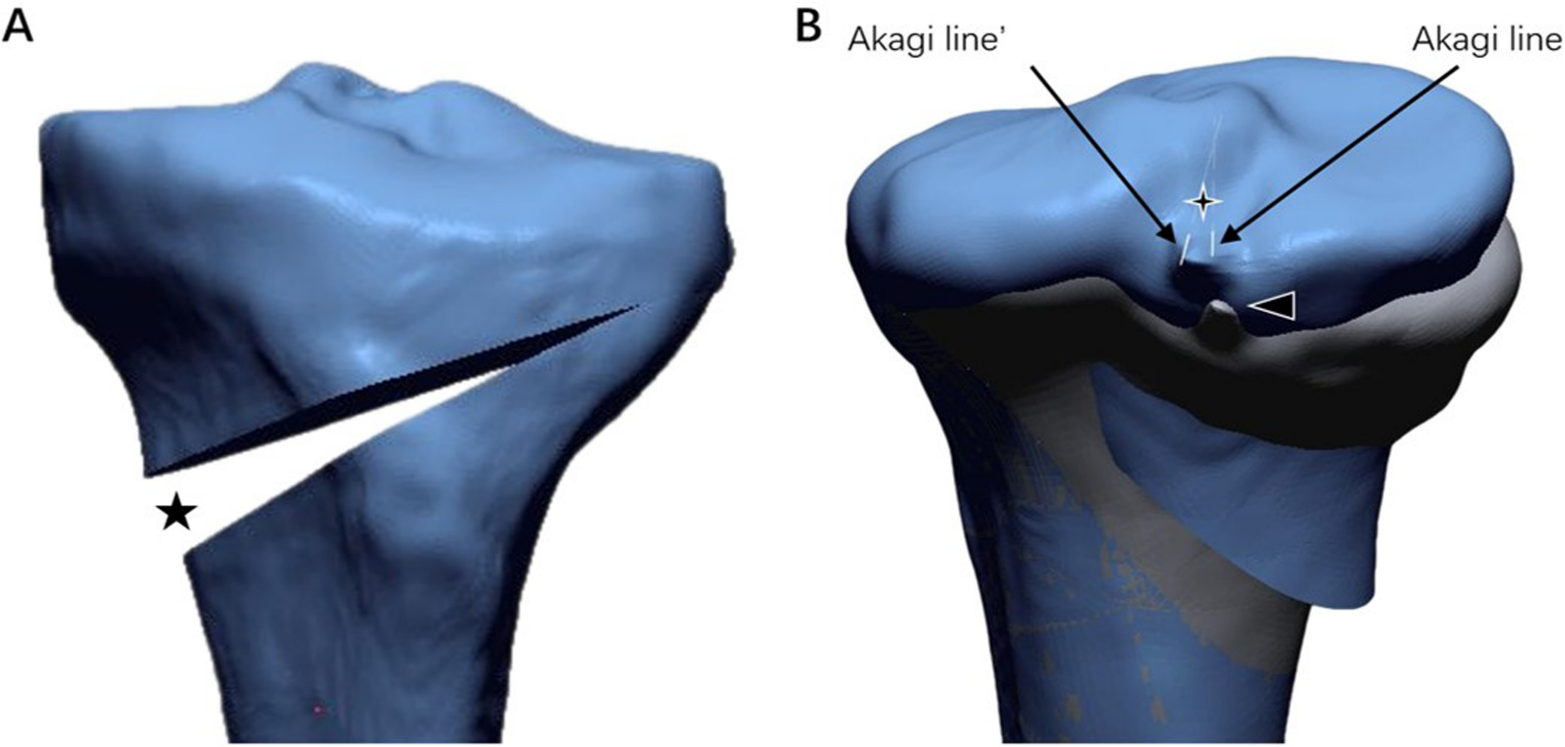
**(A)** The black five-pointed star demonstrate coronal correction angle. **(B)** The four-pointed star, angle formed by Akagi line (pre-osteotomy) and Akagi line’ (post-osteotomy), demonstrate tibial rotation. The black triangle represents the middle of the PCL.

A middle plane was created between the XZ plane and the plane parallel to the XZ plane, tangent to the interior edge of medial tibial plateau. The line through the anterior and posterior cortical edge of medial tibial plateau in the middle plane was defined as the medial tibial slope line. The hinge point was located at the tip of the fibular head level and 5 mm medial to the lateral cortex of tibia. The standard hinge position (0°) was defined as the hinge axis through the hinge point and parallel to the medial tibial slope line in the sagittal plane and Akagi line in the axial plane. A total of six hinge positions in the axial plane (±5°, ±10°, ±15°) were defined by rotating the hinge axis internally and externally around the midpoint of the standard hinge axis in the axial plane by different angles. Positive angles indicated that the front aspect of the hinge axis rotated externally, while negative angles indicated internal rotation. Similarly, six hinge positions in the sagittal plane (±5°, ±10°, ±15°) were defined by rotating the hinge axis proximally and distally around the midpoint of the standard hinge axis in the sagittal plane by different angles. Positive angles indicated that the front aspect of the hinge axis rotated distally, while negative angles indicated proximal rotation (Figures 3A,B). The osteotomy plane passed immediately over the tibial tubercle to obtain a uniplanar osteotomy. In order to reduce the measuring error of tibial rotation, a sharp protrusion at the position of the middle of the PCL was highlighted manually before virtual osteotomy (Figure 4).

### 2.5 Measurement result evaluation

The preoperative MPTA, coronal correction angle and the axial rotation of distal tibia relative to the proximal tibia during MOWHTO were recorded and evaluated. The correction angle of each patient was generated using Miniaci's method (12) and Fujisawa's point by means of preoperative standing hip-to-ankle AP radiography (13). The method of measuring rotational change in Geomagic Design X software was as follows: preoperative and postoperative 3D tibial models were overlaid manually; Akagi's lines of preoperative and postoperative 3D tibial models were drawn and projected to the XY plane; and the angles between the two Akagi's lines were measured, being defined as the rotational changes at the osteotomy site for each change in hinge axis position (14). Two researchers were trained before measurement.

### 2.6 Statistical analysis

SPSS 26.0 were used for analyses in the present study. All nominal variables were presented with frequencies and all continuous variables were expressed as means ± standard deviation. The sample size calculation was conducted using G*Power 3.1.9. 7, the minimum number of patients needed to obtain power of 0.90 in this study was 21 (effect size 0.25, *α* = 0.05). To reduce error due to inter-observer changes, all measurements were taken twice by 2 researchers independently. Intra-group correlation coefficients (ICC) were used to verify the reliability of all results. One-Way Repeated Measures ANOVA was used to compare tibial rotation changes between different groups of hinge positions in the sagittal and axial planes. If there was statistically significant differences between the groups, the single factor linear regression analysis was used to analyze the correlation between tibial rotation and hinge position in the sagittal or axial planes. *P* < 0.05 was considered statistically significant.

## 3 Results

A total of 30 knee joints in 30 patients were included. Demographic characteristics of all patients are presented in Table 1. The DTR in standard hinge position (0°), 6 axial planes (±5°, ±10°, ±15°), and 6 sagittal planes (±5°, ±10°, ±15°) hinge positions are shown in Table 2. The ICC values ranged from 0.70 to 0.95, indicating the good reliability of the measurements.

**Table 1:** Participants’ demographics and baseline characteristics**.**

Characteristic	Outcome: *N* or Mean ± SD (Range)
Numbers	30
Sex (female/male)	21/9
Side (right/left)	12/18
Age (year)	35.4 ± 10.5 (22–63)
Height (cm)	164.33 ± 8.78 (152–188)
Weight (kg)	61.09 ± 11.80 (47–93)
BMI (kg/m^2^)	22.25 ± 3.13 (16.71–28.70)
mLDFA (°)	88.07 ± 1.48 (84.75–90.90)
Pre-MPTA (°)	80.69 ± 1.91 (74.31–82.60)
Pre-aFTA (°)	181.14 ± 2.28 (177.74–186.25)
Correction angle (°)	10.59 ± 2.40 (5.34–17.87)

BMI, body mass index; mLDFA, mechanical lateral distal femoral angle; MPTA, medial proximal tibial angle; aFTA, anatomical femoral tibial angle.

**Table 2:** Tibial rotation in different groups (mean ± SD°).

Parameter	Outcome	*P* value	Parameter	Outcome	*P* value
Standard group	0.52 ± 0.73	–			
Sagittal 5 group	−0.01 ± 0.68	<0.001	Axial 5 group	0.47 ± 0.77	0.43
Sagittal 10 group	−0.61 ± 0.65	<0.001	Axial 10 group	0.52 ± 0.81	0.98
Sagittal 15 group	−1.17 ± 0.82	<0.001	Axial 15 group	0.54 ± 0.74	0.78
Sagittal −5 group	105 ± 0.84	<0.001	Axial −5 group	0.49 ± 0.79	0.66
Sagittal −10 group	148 ± 0.89	<0.001	Axial −10 group	0.53 ± 0.80	0.91
Sagittal −15 group	212 ± 1.09	<0.001	Axial −15 group	0.51 ± 0.82	0.86

*P* value, tibial rotation compared with that in standard group.

**Figure 5:**
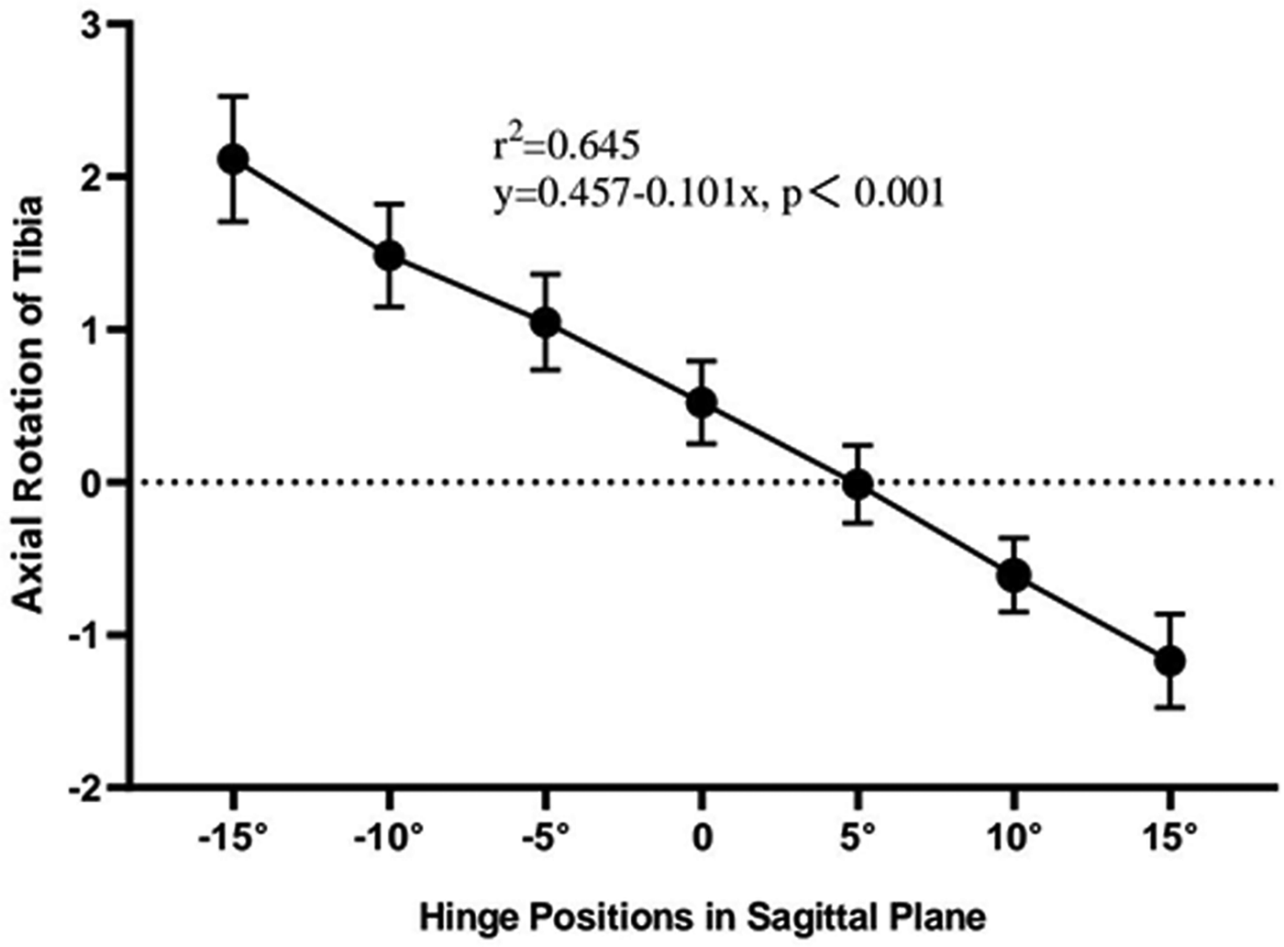
The effect of different hinge positions in sagittal plane on the change in tibial rotation. There were strong linear correlations between tibial rotation and each hinge position change. Positive values on the *y* axis represent external rotation of distal tibial, whereas negative values on the *y* axis represent internal rotation.

There were strong linear correlations between DTR and each hinge position change in the sagittal plane (Figure 5). The change in DTR was smallest when the hinge position was at 5° in the sagittal plane, −0.01 ± 0.68°, and internal or external rotation of the distal tibia may occur at such hinge position (*P* < 0.001). When the front aspect of hinge axis rotated distally, the rotations of the distal tibias of all the models tended towards internal, −0.61 ± 0.65° and −1.17 ± 0.82° when the hinge axis was at 10° and 15° in the sagittal plane, respectively (*P* < 0.001). Moreover, each 5° hinge axis rotation would result in an increase in the tibial rotation by about 0.5° internally. Meanwhile, when the front aspect of hinge axis rotated proximally, the rotation of the distal tibia tended towards external, 0.52° ± 0.73°, 1.05° ± 0.84°, 1.48° ± 0.89° and 2.12° ± 1.09° when the hinge axis was at 0°, −5°, −10° and −15° in the sagittal plane, respectively (*P* < 0.001). Similarly, each 5° hinge axis rotation would result in an increase in the tibial rotation by about 0.5° externally.

There were no correlations with each hinge position change in the axial plane when compared with that in standard hinge position (*P* > 0.5). The average tibial rotation was always about 0.5° externally in different hinge positions in the axial plane.

## 4 Discussion

The most significant finding in the present study is that sagittal hinge position changes can influence DTR, while axial hinge position changes cannot. Besides, each 5° hinge axis rotation in sagittal plane would result in 0.5° DTR change.

The direction and extent of tibial rotation during MOWHTO have been controversial, but only a few studies have reported on such issues. In clinical settings, Jang et al. (15) observed a wide range of DTR after biplane MOWHTO, from 9.5° externally to 20.9° internally. Li et al. (16) reported DTR with a range of −9.6° to +2.8° after uniplane MOWHTO in 41 knees. In a study involving 60 knees, Sasaki et al. (17) found that 32% had internal rotations, 67% had external rotations, and 1% had no rotation. In our previous study (8), a total of 106 knee joints were included, being 60% cases internal rotation of distal tibia, 38% external rotation, and 2% no rotation. Some researchers explored DTR during MOWHTO using cadaver limbs as well (18, 19). A study by Jacobi et al. (18) demonstrated that in 11 cadaver limbs, the average rotation change of distal tibia was 1.5° ± 2.9° internally with a range of 4.5° external rotation to 15° internal rotation. A study by Kendoff et al. (19) demonstrated that in 13 cadavers, the distal tibia was externally rotated in 10 of the 13 cadavers and the mean rotation increased by 2° ± 6°, ranging from a maximal internal rotation of 9° to a maximal external rotation of 12°. By means of virtual osteotomy with 3D tibial models, we found external, unchanged and internal rotation may occur and more cases tended to internal rotation as mentioned above. Besides, each 5° hinge axis rotation in sagittal plane would result in 0.5° DTR change, which has not been reported in the previous literature.

The related influencing factors of tibial rotation have been investigated by several researchers, but an uncontested result has yet to be provided. Clinically, Kim et al. (4) found that the degree of DTR was not related to the amount of coronal correction after MOWHTO. However, Kim et al. (20) reported that the correction angle was the only predictor of DTR, with a larger correction angle observed in the group with DTR >3° compared to the group with rotation ≤3°. As far as hinge axis position was concerned, Lee et al. (14) found a positive correlation between the hinge axis angle and internal rotation of the distal tibia when the hinge axis was positioned more posterolaterally. In our previous study (8), DTR change was significantly associated with angles between standard and actual hinge in the sagittal planes (ASAHS), followed by opening width and flange angle. In a cadaveric study conducted by Jacobi et al. (18), advanced OA with concomitant flexion contracture was found to be associated with a significantly decreased tibial rotation change. In a 3D surgical simulation study by Chang et al. (7), a large angle of wedge inclination, which was the incline angle of the bone saw relative to the plateau plane, was found to be an indicator of horizontal rotation. The aim of Chang's study was partially similar to that of the present study, but obviously, the deficiency of his study was that the sample size was too small, only a proximal tibia of a healthy subject was reconstructed. In our study, through further research with virtual osteotomy involving 30 cases, we found that it is sagittal but not axial hinge axis affects the direction and extent of tibial rotation. The virtual osteotomy on a 3D model can eliminate some unknown confounding variable and allow for a more focused investigation of the bone structure, making the conclusion more reliable.

Several scholars have reported that DTR can affect TT-TG distance (21, 22). Cheng (21) included 69 patients undergoing MOWHTO, and found that the TT-TG distance decreased by an average of 3 mm postoperatively, with the authors attributing the main reason to the tibial tuberosity shift medially caused by the internal rotation of the distal part of the osteotomy. Similarly, Sim (22) found that the average TT-TG distance decreased by about 2 mm postoperatively, with the authors also speculating that it was related to the internal rotation of the distal part of the osteotomy. Large TT-TG distance has an adverse effect on the patellofemoral joint (23–25). Otsuki (26) analyzed 85 patients with knee varus found that patients with larger TT-TG distance had more severe patellofemoral arthritis on CT scans. Huntington (24) found elevated TT-TG distance was a key risk factor for the recurrence of lateral patellar dislocations. Combined with the present study, positioning the osteotomy hinge as anteriorly inclined as possible in the sagittal plane is a practical measure to prevent external rotation of the distal part of the osteotomy to minimize its impact on TT-TG distance during MOWHTO.

There are some limitations in the present study. First, there was a relatively small sample size with only 30 models. Second, the uniplanar osteotomy performed is different from biplanar osteotomy which is more popular in clinical practice. It is generally believed that an ascending osteotomy plane would have an significant effect on DTR in the biplanar osteotomy, but as mentioned in our previous study, DTR change was significantly associated with ASAHS, followed by correction angle and ascending osteotomy plane if ranking osseous factors by their effects on DTR during biplanar MOWHTO (8). Furthermore, considering uniplanar MOWHTO being reported by some literatures (16, 27–29), undeniably it was very meaningful for this study. Third, the present study is a virtual surgical study conducted using software, and the soft tissue around osteotomy site or the position of plate were not considered, which may not fully replicate clinical conditions encountered during MOWHTO. Additional research aimed at clinical validation along with clinical outcomes such as the influence on articular cartilage and function will be of interest. However, to the best of our knowledge, there has been no 3D simulation MOWHTO study focusing on tibial rotation yet, and further investigations with a larger sample size and clinical researches are needed.

## 5 Conclusion

It is sagittal but not axial hinge axis affects DTR in uniplanar MOWHTO with three-dimensional tibial models. In the sagittal plane, every change in hinge position was significantly linearly correlated with DTR. However, no linear correlations were observed between every hinge position change in the axial plane. Besides, each 5° hinge axis rotation in sagittal plane would result in 0.5° DTR change.

## Data Availability

The raw data supporting the conclusions of this article will be made available by the authors, without undue reservation.
